# Pills and prayers: a comparative qualitative study of community conceptualisations of pre-eclampsia and pluralistic care in Ethiopia, Haiti and Zimbabwe

**DOI:** 10.1186/s12884-021-04186-6

**Published:** 2021-10-26

**Authors:** Tanya Robbins, Charlotte Hanlon, Ann H. Kelly, Muchabayiwa Francis Gidiri, Mickias Musiyiwa, Sergio A. Silverio, Andrew H. Shennan, Jane Sandall

**Affiliations:** 1 Department of Women & Children’s Health, School of Life Course Sciences, King’s College London, St Thomas’ Hospital, 10th Floor, North Wing, Westminster Bridge Road, London, SE1 7EH UK; 2 Centre for Global Mental Health, Health Service and Population Research Department, and WHO Collaborating Centre for Mental Health Research and Training, Institute of Psychiatry, Psychology and Neuroscience, King’s College London, London, UK; 3 Department of Psychiatry, WHO Collaborating Centre for Mental Health Research and Capacity-Building, School of Medicine, College of Health Sciences, Addis Ababa University, Addis Ababa, Ethiopia; 4 Centre for Innovative Drug Development and Therapeutic Trials for Africa (CDT-Africa), College of Health Sciences, Addis Ababa University, Addis Ababa, Ethiopia; 5 Department of Global Health and Social Medicine, King’s College London, London, UK; 6 Department of Obstetrics and Gynaecology, College of Health Sciences, University of Zimbabwe, Harare, Zimbabwe; 7 Department of History, Heritage and Knowledge Systems, Faculty of Arts and Humanities, University of Zimbabwe, Harare, Zimbabwe

**Keywords:** Pre-eclampsia, Hypertensive disorders in pregnancy, Ethiopia, Haiti, Zimbabwe, Delays in care-seeking, Pluralism, Decision-making in pregnancy

## Abstract

**Background:**

Pre-eclampsia is a leading cause of preventable maternal and perinatal deaths globally. While health inequities remain stark, removing financial or structural barriers to care does not necessarily improve uptake of life-saving treatment. Building on existing literature elaborating the sociocultural contexts that shape behaviours around pregnancy and childbirth can identify nuanced influences relating to pre-eclampsia care.

**Methods:**

We conducted a cross-cultural comparative study exploring lived experiences and understanding of pre-eclampsia in Ethiopia, Haiti and Zimbabwe. Our primary objective was to examine what local understandings of pre-eclampsia might be shared between these three under-resourced settings despite their considerable sociocultural differences. Between August 2018 and January 2020, we conducted 89 in-depth interviews with individuals and 17 focus group discussions (*n* = 106). We purposively sampled perinatal women, survivors of pre-eclampsia, families of deceased women, partners, older male and female decision-makers, traditional birth attendants, religious and traditional healers, community health workers and facility-based health professionals. Template analysis was conducted to facilitate cross-country comparison drawing on Social Learning Theory and the Health Belief Model.

**Results:**

Survivors of pre-eclampsia spoke of their uncertainty regarding symptoms and diagnosis. A lack of shared language challenged coherence in interpretations of illness related to pre-eclampsia. Across settings, raised blood pressure in pregnancy was often attributed to psychosocial distress and dietary factors, and eclampsia linked to spiritual manifestations. Pluralistic care was driven by attribution of causes, social norms and expectations relating to alternative care and trust in biomedicine across all three settings. Divergence across the contexts centred around nuances in religious or traditional practices relating to maternal health and pregnancy.

**Conclusions:**

Engaging faith and traditional caregivers and the wider community offers opportunities to move towards coherent conceptualisations of pre-eclampsia, and hence greater access to potentially life-saving care.

**Supplementary Information:**

The online version contains supplementary material available at 10.1186/s12884-021-04186-6.

## Background

Maternal health inequities remain an urgent global concern despite improvements over recent decades [1]. Pre-eclampsia is a leading cause of maternal and perinatal mortality and morbidity in under-resourced settings [2, 3]. A complex and variable pregnancy-specific, multisystem disease, the aetiology of pre-eclampsia remains to be fully elucidated [4]. Current evidence suggests pre-eclampsia is a multistage disorder involving abnormal placental development, which occurs in a pre-clinical, symptomless stage [5]. Pre-eclampsia can be unheralded and unpredictable, contributing to uncertainty for women and care providers. Pre-eclampsia can present during pregnancy, delivery or in the postnatal period. Women may develop headache, visual disturbance, abdominal pain, significant swelling including of the hands and face, or may be asymptomatic. Furthermore, symptoms do not necessarily correlate with severity and deterioration can be rapidly progressive.

Pre-eclampsia is diagnosed by the presence of new onset hypertension after 20 weeks’ gestation with evidence of other end organ involvement [6]. Severe complications due to pre-eclampsia can occur without adequate monitoring and treatment. These include eclamptic seizures, stroke and organ dysfunction as well as fetal growth restriction and stillbirth. One of the main objectives of antenatal care is to screen for pre-eclampsia with regular monitoring of blood pressure, urine and fetal growth [7]. Effective management involves early recognition and control of raised blood pressure, prevention or treatment of seizures and timing of delivery [6, 8–11]. Medications to treat hypertension or seizures do not halt disease progression but delivery of the placenta, and therefore the baby, initiates resolution of the disease process. Early delivery, to reduce the risk of maternal complications associated with continuing pregnancy, must be carefully balanced against the risks to the baby, associated with prematurity. Current evidence for optimal timing of delivery in under-resourced settings is scarce, but ongoing trials are being conducted [12]. Contextual challenges related to timing delivery, specific to low- and middle-income countries (LMICs) include accurate dating of pregnancy and the limited availability of specialist neonatal care.

The seminal framework set out by Thaddeus and Maine (1994) describing delays contributing to poor obstetric outcomes, considered that factors beyond distance, cost and quality of care play into decision-making processes around maternal health. Healthcare seeking behaviour is strongly influenced by the characteristics of the illness as perceived by the individual [13]. A study of participatory women’s groups in Malawi found hypertension in pregnancy or seizures were not amongst the five problems prioritised as most important [14]. Previous qualitative and quantitative studies have investigated community knowledge of pre-eclampsia in a variety of LMICs [15–22]. A limited understanding of warning signs, symptoms and outcomes associated with pre-eclampsia and eclampsia has been reported and local perceptions were noted not to align with biomedical perspectives. Pre-eclampsia has been attributed to spiritual, emotional and social factors [19, 20, 23]. However, previous studies have tended to focus on a single country and include limited community perspectives without involving health professionals. A study of survivor perceptions of pre-eclampsia in Nigeria recommended future research should explore interpersonal relationships, social norms and further probing of spirituality to understand nuanced influences on care-seeking and decision-making [20]. Previous work has suggested a need to increase awareness and dispel common myths. However, approaches that seek to disabuse people of their understanding often close down the possibilities of engaging and exploring different perspectives. There are very few studies published on health education specifically targeting pre-eclampsia in low resource settings. The existing literature has limited descriptions of contextual barriers to care or of the intervention development processes.

This comparative cross-cultural study explores local understanding, lived experience and knowledge that influences care for women with pre-eclampsia in Ethiopia, Haiti and Zimbabwe. The study seeks to deepen our understanding of how illness related to pre-eclampsia is interpretated by individuals and communities. We explore subjective meanings and actions relating to pre-eclampsia across a broad range of perspectives. Examining to what extent these diverge or converge across settings will help to further understand how cultural contexts around pregnancy shape understanding and prioritisation in relation to where and when to seek care.

## Methods

### Study settings

This study was conducted in Ethiopia, Haiti and Zimbabwe. All three countries have similarly high maternal mortality ratios (MMR), although rates have been falling in Ethiopia while they remain almost static in Haiti and Zimbabwe. (Table 1) The Ethiopian study area included Meskan and Sodo districts in the Gurage Zone, Southern Nations, Nationalities and People’s Region (SNNPR) approximately 130 km south of Addis Ababa. In Haiti, the study was conducted around the coastal city of Cap-Haitian within the Northern Region, 240 km from the capital Port-au-Prince. Zimbabwean study sites included Tafara and Mabvuku, high density suburbs of Harare, and peri-urban Makumbe 50 km away. Across settings, pregnancy, birth and the postnatal period are associated with a heightened vulnerability to emotional and spiritual harm [24]. Traditional health systems include an eclectic mix of herbalists, prophets and sorcerers, as well as traditional birth attendants (TBAs), some of whom are faith-based. These diverse settings were selected to allow comparison of findings across distinct political, economic and sociocultural contexts.

**Table 1:** National level maternal and neonatal context

Health and Development Indicators	Ethiopia	Haiti	Zimbabwe
Maternal mortality ratio (deaths per 100,000 live births)^a^	401 (2017) 1030 (2000)	480 (2017) 437 (2000)	458 (2017) 579 (2000)
Stillbirth rate (per 1000 total births) (2015) ^a^	30	25	21
Antenatal care (4+ visits) ^a^	43% (2019)	67% (2017)	72% (2019)
Delivery with skilled birth attendant^a^	55%	37%	89%
Institutional delivery^a^	26% (2016)	39% (2016)	85% (2019)
Eclampsia rates (per 10,000 deliveries) [25]	57.3	83.8	56.8
Adolescent birth rate (births per 1000 girls 15–19 years) (2019)+	66.7	51.7	86.1
Total fertility rate (births per woman) (2019) ^a^	4.1	2.9	3.5
Secondary education completion rate (females) in 2018^a^	13%	16%	11%
Total population (2019) ^a^	112,079,730	11,263,077	14,645,468
Urban population^a^	21%	56%	32%
GDP per capita (USD $) (2019) ^a^	855	1272	1464
Percentage GDP spent on health (2018) ^a^	3.3	7.69	4.73
Human Development Index rank (2019)^b^	173	170	150

^a^Source: UN MMEIG 2019 (United Nations Maternal Mortality Estimation Inter-agency Group)

https://data.worldbank.org/indicator/SH.STA.MMRT

^b^Source: United Nations Development Programme, Human Development Reports

http://hdr.undp.org/en/indicators/36806#b

[25]Source: Vousden, N., et al. (2019). “Incidence of eclampsia and related complications across 10 low- and middle-resource geographical regions: Secondary analysis of a cluster randomised controlled trial.” PLoS Med **16** (3): e1002775

#### Ethiopia

Ethiopia is a diverse country with a population made up of over 80 ethnic groups, who are predominantly rural dwellers. The official language is Amharic. Ethiopia has a distinctive political and social history, having never been under colonial rule. The main religions are Ethiopian Orthodox Christianity, Islam and Protestant Christianity. Holy water (*tsebel)* and prayer are commonly used for faith healing. The current government-run biomedical health system is structured into three tiers. At primary level, the primary health care unit consists of a health centre and below this there are health posts. Health posts are staffed by two health extension workers (HEWs). The Health Extension Programme, established in 2004 introduced this new (paid) cadre, trained to deliver a package of basic health services in rural areas, including maternal care and health education [26]. One of the roles of HEWs is to train ‘model families’ in their neighbourhood, to demonstrate good health behaviours and influence their community. The programme now includes the Women’s Development Group (WDG). The WDG is an organised volunteer movement designed to improve engagement and mobilise communities. Integrated emergency surgical officers (IESO) were introduced as part of task shifting efforts to reduce mortality. They are responsible for managing many of the common obstetric emergencies at secondary care level.

#### Haiti

Vast inequality exists in Haiti between the Creole-speaking black majority and the more affluent French-speaking Haitians of mixed African and European descent. Haiti has suffered political, economic and environmental instability and is the poorest country in the Western Hemisphere. Following the devastating earthquake in 2010, Haiti saw an influx of international aid organisations and foreign actors each with their own priorities and protocols. 42% of Haiti’s health institutions are private, 38% public and 20% mixed [27]. There is poor coordination between the non-profit and for-profit private sector, and public health services. Many women deliver with TBAs, some linked to community health workers (CHW) working alongside facilities within the formal health system, who may have received some basic training in the past, and others with no formal training who often learn through experience. Catholicism is the predominant religion and approximately 20% of the population are Protestants. Different religious and healing traditions are intertwined in Haiti. Many Haitians identifying as Christians are also influenced by Vodou beliefs that are deeply woven into cultural life and folklore [28]. The Vodou religion originating from West Africa, also draws on Taìno Indian religious practices and centres around the worship of spirits (*lwa*). The spirits are believed to preside over different aspects of earthly existence and communicate with humans during religious rituals. Spirits are understood to inhabit a person’s body during possession, often manifesting as a seizure. Vodou priests or healers are held to possess supernatural powers that enable communication with the spirits. Much of the success of the Haitian revolution was attributed to the gods, a source of hope and motivation [29].

#### Zimbabwe

Shona, Ndebele and English are the most widely spoken languages in Zimbabwe. Chronic political and economic instability have taken their toll on Zimbabwe’s health sector. A once thriving national health system now suffers with shortages of basic supplies and disaffected medical professionals struggling to deliver services [30, 31]. Following Zimbabwean independence, the government recognised TBAs and supported training. Following shifts in policy toward skilled birth attendance, TBA practice now continues outside the formal health system. However, institutional delivery rates are relatively high compared to the other countries. The main religious influences in Zimbabwe are African traditional religions and Christianity. Some conservative Apostolic churches have strict religious regulations and object to modern healthcare practices [32]. Spiritual influences within the Apostolic faith focus on the Holy Spirit (*Mweya Mutsvene*) and ancestral or evil spirits (*mweya yetsvina)* that can negatively affect health. *Mweya* foretells illness, tragedy or complications but also offers treatments and works through prophets endowed with healing and prophetic powers. Prophets can be used to promote maternal and child health, cleansing evil spirits through rituals including the use of holy water, prayers, sanctified stones and herbs.

### Data sampling and collection

We undertook a qualitative study using semi-structured face-to-face in-depth interviews (IDIs) and focus group discussions (FGDs). Our local partners included researchers from Addis Ababa University, the University of Zimbabwe and a non-governmental organisation supporting a faith-based general hospital in Haiti. This study was nested within two wider projects: The HAPPEE (Humanities and Arts in Preventing Pre-eclampsia complications through community Engagement and Education) Partnership Project in Haiti and Zimbabwe, and ASSET (Health System Strengthening in sub-Saharan Africa) in Ethiopia [33]. In order to elicit diverse perspectives, local facilitators purposively sampled a broad range of participants likely to be influential in maternal care. The constituents varied across the countries reflecting local contexts and many were sampled via community health workers or local health facilities. We included pregnant and postnatal women, survivors of pre-eclampsia, families of women who had died due to pre-eclampsia, partners, older male and female decision-makers (mothers and fathers-in-law), religious leaders and healers, traditional healers, traditional birth attendants, community health workers (including HEWs and WDG in Ethiopia) and facility-based health professionals. (Table 2) Interviews and FGDs were conducted in local languages and led by researchers with prior experience of qualitative data collection. Written participant information sheets were read out. All participants were required to indicate informed consent, either through thumbprint or signature. The local research teams included a female community auxiliary nurse in Haiti; a female research coordinator with a background in midwifery, a male obstetrician and a male professor of arts and knowledge systems in Zimbabwe; and a female social epidemiology research coordinator in Ethiopia. A female British obstetrician (TR) attended most interviews and FGDs in Haiti and Zimbabwe. Data were collected between August 2018 and January 2020. Topic guides were developed in collaboration with local researchers and included questions on caring for pregnant women, complications faced and experiences of high blood pressure during pregnancy. Discussions were audio-recorded, transcribed and translated into English and a sub-sample cross-checked for accuracy by local co-investigators. Supplementary notes and a reflexive diary were also taken by TR and triangulated with transcripts. Data collection took place in health facilities, community spaces or participants homes.

**Table 2:** Participant characteristics

Interviews (IDI)	Ethiopia	Haiti	Zimbabwe
Pregnant women	6	–	–
Women with previous pre-eclampsia	8	3	2
Family of deceased women	4	–	2
TBA	4	1	1
WDG	4	–	–
CHW/HEW	6	2	–
Community leaders	3	–	–
Midwife/nurses	6	2	5
Doctors	3	2	3
Integrated emergency surgical officers (IESO)	3	–	–
District health manager	1	–	–
Religious leaders	–	1	8
Faith healers	–	–	4
Traditional healers	–	2	3
**Focus group discussions (FGD)**			
Pregnant women	–	2 (*n* = 10)	2 (*n* = 12)
Postnatal women	–	1 (*n* = 6)	2 (*n* = 11)
Older female decision-makers (mothers/mother-in-law)	–	1 (*n* = 3)	2 (*n* = 9)
Male decision-makers (partners/husbands/fathers/fathers-in-law)	–	1 (*n* = 6)	2 (*n* = 10)
TBAs	–	1 (*n* = 8)	1 (*n* = 6)
CHW	–	–	1 (*n* = 6)
Community leaders	–	1 (*n* = 7)	–

### Data analysis and theoretical underpinning

In order to facilitate comparison across countries and between participant groups, we conducted template analysis, a form of thematic analysis [34]. Following data preparation and familiarisation, a preliminary template was developed, drawing on a combination of Social Learning Theory (SLT) and the Health Belief Model (HBM) [35]. These approaches were combined to consider different levels of influence on maternal health behaviour. SLT understands human behaviour as governed by a triangulation of cognitive, behavioural and environmental factors including relational components such as social norms. The HBM centres on intrapersonal behaviour. It hypothesises that an individual will take health related action if they believe they are susceptible to a condition, if they have knowledge of the severity or consequences of the condition, and if the perceived benefits outweigh perceived barriers. The template was discussed and refined with co-investigators (Additional file 1). Using NVivo 12 software, all data were coded into the template by TR and a sub-sample cross-checked and discussed with SAS. The initial template was adapted, allowing renaming and reordering of codes as indicated by the data. Data were charted and findings summarised with illustrative verbatim quotations from each transcript. Participants were collated into three broad analysis groups comprising women of reproductive age, the wider community and facility-based healthcare professionals. Further interviews were conducted where data saturation was not reached including a wider sample of religious leaders in Zimbabwe and women who had experienced pre-eclampsia in Ethiopia*.* Themes were iteratively developed, reviewed and defined around central concepts where data were richest. Member checking was undertaken via Theory of Change workshops in later stages of the wider projects described earlier, which involved summarising and presenting our findings back to participants.

#### Findings

Participants across all three countries and analysis groups recognised pregnancy and childbirth as a time of increased susceptibility to problems, including the risks of mothers and babies dying. Headache, visual disturbance and swelling were mentioned by many as indicators of potential problems. However, a lack of community knowledge of specific complications, such as high blood pressure during pregnancy, was also described. This included women who had previously suffered with pre-eclampsia and life-threatening complications such as eclampsia. Many survivors of pre-eclampsia reported being unsure of the meaning of their experiences and not connecting them to risks associated with pre-eclampsia. For some women urgent help-seeking was not triggered until in extremis. We found that symptoms and problems associated with pre-eclampsia were interpreted in different ways within and across settings. We present three main themes around conceptualisations of illness related to pre-eclampsia, how complex societal forces shaped care-seeking and opportunities for inclusivity to improve care.

### Theme I. Challenges in coherence: a lack of shared language and interpretations

Pre-eclampsia was reported to be unpredictable and variable in how and when it presented. Respondents noted difficulties recognising a condition that may have no obvious manifestations. When problems related to pre-eclampsia became overt, they were described as sometimes subtle and intermittent such as reduced fetal movements, or extreme and vivid as in the case of eclampsia. Participants spoke of uncertainty, inconsistency and precarity which affected their trust and acceptance of a diagnosis and subsequent advice. These feelings made conceptualising pre-eclampsia challenging. Seemingly contradictory realities were difficult to make sense of as a collective idea of illness.

*“I had a check-up on a Thursday, and I was told that it was 70 [blood pressure]. So, when I was told I was hypertensive, I did not believe them because just the last Thursday, I was normal. Yes, I didn’t believe them, so I went out to seek a second opinion.”*

(Woman with previous pre-eclampsia, Ethiopia, M25)

*“Yes, after the blood pressure happened, the disease does not give you any sense inside, you know that, because it might be giving sense since I did not know. I thought of the pregnancy as the only disease which gives me stress. It is only about the pregnancy that I know, nothing else. I mean it came without my consent and was gone without my consent.”*

(Woman with previous pre-eclampsia, Ethiopia, M28).

Language presented another challenge to coherence in conceptualisations of pre-eclampsia. In many local languages a direct translation of pre-eclampsia was not possible. There were not words to describe this complex, pregnancy-specific condition as defined in a biomedical sense. Although in some communities the condition may not exist as a coherent idea of an illness, the manifestations were translated in various descriptive or interpretive terms related to the phenomena to which they are attributed. Descriptive terms include direct translations of afflictions observed; for example, in cases of eclampsia in Ethiopia, tremoring, shivering or loss of consciousness may be used interchangeably. There is no Shona translation for hypertension. *BP* (referring to blood pressure) is widely used to describe high blood pressure, either during or outside pregnancy. In Zimbabwe, interpretive terms, such as *chikandwa,* may be used to explain high blood pressure in pregnancy where it’s attributed to spirits. Literally translated, *chikandwa* is something someone has thrown on another person, referring to an act of witchcraft. Epilepsy (*pfari/tsviyo),* seizures (*kugwinha)* and frothing at the mouth (*kubuda furo*) are used to describe eclampsia. Where terms more akin to a biomedical paradigm do exist, interpretations still vary. In Haitian Creole, there is a direct translation of the word eclampsia (*eklampsi)* which is used to describe a condition where convulsions occur during pregnancy. The distinction between pre-eclampsia and eclampsia is not always clear in Haiti. *Eklampsi* may be used interchangeably to describe pre-eclampsia, for example a woman presenting with severe swelling that is commonly understood to be linked to high blood pressure. *Kriz* (seizures) and *move lespri (*bad spirits) were other Creole words used to describe eclampsia. Different perceptions are illustrated by the various terms used to describe manifestations of pre-eclampsia and are often linked to their attributions.

*“If you become sick of anything, if you go to the doctor, he will find a name for it, right? Even if you get bewitched.”*

(Apostolic religious leader, Zimbabwe, IDI28)

In all three settings, women and communities commonly attributed high blood pressure in pregnancy to diet, including excess salt, sugar, spicy food, coffee or oil, and to states of emotional distress. These perceptions were shared by healthcare professionals, leading to counselling centring around these factors.

*“Well, if they tell you not to eat salty or spicy foods, sodas, maybe that’s what causes your blood pressure to go high before and after having the baby.”*

(Antenatal woman, Haiti, FGD3)

*“Yes, we tell her to reduce stress or not to be stressed and restrict salt.”*

(Midwife, Ethiopia, M5)

Attributions of hypertension in pregnancy to emotional distress arising from social stressors included marital conflict, abuse, difficult wider family relationships, poor living conditions or financial worries. These were described to lead to “too much thinking”, bitterness and pregnant women feeling angry. In some instances, these were linked to manifestations of evil or ancestral spirits understood to cause pregnancy complications, such as seizures or maternal death.

*“Ya, you know when someone dies in Shona, they say when death happens someone is blamed for it, like bewitched. When someone dies, it doesn’t matter if is the wife who dies or the husband dies, people tend to blame the other spouse, that maybe there was conflict in the marriage…we have three children all girls, so some people were saying that we heard that you are tormenting your wife, because you want a boy. That is why she had raised blood pressure, of which it’s something that never happened, nothing like that happened.”*

(Husband of deceased woman, Zimbabwe, IDI27)

*“Yes, I know blood pressure resulted from being angry, stressed, and I am convinced about this. I believe that it was from anger and stress, therefore, I did not want her to be angry and stressed.”*

(Family of deceased woman, Ethiopia, M26)

Less commonly, pre-eclampsia was understood to be linked directly to the pregnancy, distinct from other hypertensive disorders and observed to resolve following delivery.

*“It is caused because of the pregnancy…It comes when I am pregnant. I don’t have it now.”*

(Woman with previous pre-eclampsia, Ethiopia, M24)

*“We call it high blood pressure that is related to pregnancy and there is the other one when you are normal [not pregnant] and you have high blood pressure. Those are not the same. We take them as two different items. There is high blood pressure that stops after giving birth, there is also high blood pressure that develops during pregnancy and continues even after she gives birth.”*

(HEW, Ethiopia, M3).

### Theme II. Complex priorities: balancing expectations and preferences

Women and communities negotiated multiple knowledge systems, based on their own experiences, realities, preferences and also on their wider social contexts. They availed help and advice from biomedical professionals but also from religious and traditional healers, TBAs and other community caregivers. Medical pluralism and patterns of resort were complex and grounded in social norms and expectations, as well as individual preferences. Our study found many examples of pluralistic care seeking. Attribution of causes, women’s autonomy and trust influenced where, when and in what order help was sought.

### Attribution of causes

The use of alternative care providers such as faith or traditional healers was linked to women and communities’ perceptions of causes of complications during pregnancy across all settings. Where problems were understood to be spiritual in nature, for example evil spirits or curses leading to seizures, swelling or headaches, prayers or religious care were availed. In these instances, hospital care was not perceived to be necessary. Religious care was either protective and understood to prevent the occurrence of problems or curative and used to heal once symptoms developed.

*“That’s the first thing; they want to understand what happened, but people will be knowing that the person has BP, but they are quick to look for a witch. They say ‘aah let’s first consult the prophets’, while the person will be getting worse…They will be finger pointing each other in their family…Or the mother-in-law, she can be bewitched by her mother-in-law…They will finger-point each other, instead of rushing the person to the hospital, they rush her to the prophets so that’s another risk that is”.*

(Male decision-makers, Zimbabwe, FGD10)

*“…the community has a big misunderstanding when it comes to eclampsia because it has symptoms like seizure and convulsion which help them to consider witchcraft relating it to their religion. So, they also believe that bringing that mother to the health facility will not solve a thing. This time they will try to manage the problem traditionally. I have encountered similar cases regarding this…”.*

(Midwife, Ethiopia, M33)

*“Well, it’s a ritual thing because here in our country if the person has a headache, she won’t think it’s because of a high blood pressure she mostly thinks it’s because this person or that person is doing something to harm them.”*

(Doctor, Haiti, IDI13)

*“But God created, because in our Shona culture, we say, when a person had mischief [committed adultery], she must confess it when she gives birth… That is the danger of doing things that cannot be confessed and cannot be seen by a machine [through scan] and doctors cannot bring out such an issue.”*

(Apostolic Religious Leader, Zimbabwe, IDI28)

In Haiti, prayer was perceived by women to be protective in situations involving social conflict, which were often linked to problems during pregnancy. Prayers and faith were perceived to prevail over facility-based care but were also not understood to be mutually exclusive.

*“Well, praying is good, it’s like a first care that you get because it can help you a lot.... praying is more efficient than everything else we have said, all we do is because of God. Even if you’re a doctor but God comes before everything”.*

(TBA, Haiti, IDI3)

In Zimbabwe, there were also examples of biomedical care seeking alongside alternative healing.

*“Some of the problems that we experience among pregnant women are that some mix, they would be coming to the hospital, they would be going to the traditional healers and to the prophets they will also be going.”*

(Community health workers, Zimbabwe, FGD6)

### Shared decisions and resources

Decision-making processes around maternal care were often described as joint endeavours involving husbands or partners and sometimes the wider family. In Zimbabwe, women reported that this also often involved religious leaders and mother-in-law. Women also described husbands being influenced by their parents. Some women expressed a lack of trust in their parents-in-law. The tradition of women returning to their own parental home during their first pregnancy, was thought to be protective against potential curses from their in-laws, preventing them from interfering with delivery including exerting pressure to take traditional remedies. Less commonly, women described taking decisions about their health independently. However, a commonality across the settings related to male control of financial resources necessary to seek care, and the need therefore for pregnant women to seek male support.

Other financial barriers described in relation to care for pre-eclampsia included fear of or actual out-of-pocket payments for services. Poverty was widely described as determining constraint in antenatal and emergency care trajectories, across all three countries, despite national health policy advocating for free maternity care. Contextual differences were described in relation to financial barriers to biomedical care across the settings. In Ethiopia, the hospital within the study setting required women to pay for emergency care if it was not related to delivery. This included women admitted with pre-eclampsia requiring monitoring but not delivery. In Haiti, women and community members feared hospital detention in case of non-payment of fees. In Zimbabwe, a double barrier was described, as those unable to pay to register for antenatal care were also more fearful of attending facilities for delivery or emergency care. This led to worrying about how they would be treated by staff, having not sought care earlier in pregnancy.

Alternative healing was seen by some to surmount financial barriers associated with facility-based care. Alterative caregivers were more likely to live close by, within the community, and accept lower or alternative forms of payment or to offer free services.

*“The reason they use traditional treatment is, firstly since they don’t spend money on it or since it is free and is easily accessible.”*

(Midwife, Ethiopia, M5)

### Trust and expectations

Healthcare professionals perceived that women were likely to seek alternative care because they had more trust in traditional or religious healing.

*“I don’t think they fully believe in the conventional medicine. They only come to us when their case becomes severe and they couldn’t handle it any longer. They would rather go to the traditional route.”*

(Doctor, Ethiopia, M12)

*“In my opinion what happens is a lot of people don’t think the hospital can do much for them. They believe in witch doctors. They always think that the witch doctors can do everything.”*

(Nurse, Haiti, IDI2)

Communities described complex relationships with alternative care providers. In Zimbabwe, women described the existence of ‘true’ and ‘false’ prophets or faith healers, expressing uncertainty about their efficacy or potential for religious healing. False prophets were understood to be those motivated by financial gain. Male decision-makers in Zimbabwe also perceived some prophets sought to create disharmony amongst families by prophesying bewitchment between relatives.

Some individuals expressed a preference for biomedical care as they perceived it to be beneficial. However, their responses indicated a more complex attitude to traditional medicine: acceptance of it, but not always grounded in a belief in its safety or efficacy. In describing acceptance, it was clear that utilisation of traditional approaches reflected a compliance with social norms: women were expected to accept remedies, go through the rituals; this was not always followed.

*“I’d rather go to the doctor. Doctors studied for what they are doing. The leaf might be good, but you might die. If someone gives me a leaf to make tea, I’ll take it from you. That doesn’t mean I am going to use it. Because you might see it as being rude, so I’ll take it from you.”*

(Female decision-maker, Haiti, FDG5)

Across all settings biomedical care was seen to offer potential benefits to pregnant women by many women and community members. These included access to medications, professional advice, knowledge of gestational age of the pregnancy, identification of complications such as high blood pressure, referral to higher level care if needed and a reduced risk of developing problems or dying. Positive perceptions also related to previous experience of quality care once inside the system.

*“All the patients including me at the operation room were hypertensive... They took care of us well. There were also nurses and they took care of us well...Yes, they come right away when we call them like when my headache was getting stronger.”*

(Woman with previous pre-eclampsia, Ethiopia, M25)

“*It is best for them to go to the clinic and get everything, and even avoid blood pressure. At the end of the day, it will burn the child but if you go to the clinic, they control that blood pressure.”*

(Pentecostal religious leader, Zimbabwe, IDI17)

*“I went to the hospital and they gave me pills, they checked me out. I had to do a lot of tests. And I felt better.”*

(Antenatal woman, Haiti, FGD3)

### Theme III. Opportunities for inclusivity, improving quality and support

Biomedical professionals emphasised respect for the relationship between their patients and faith, acknowledging and appreciating its role in their lives as well as a need to educate religious communities on seeking timely facility care. They described respecting and understanding the practices passed down through families. In Zimbabwe, a midwife recounted an opportunity to engage with the religious leader of an Apostolic sect within which women were often forbidden from seeking maternal care at health facilities.

*“The Apostolic sect, you know they used to do their own things. So, on this particular day, this lady came eclamptic at their church, they thought she was possessed with demons. They spent the whole night with her praying, trying to cast out the demon in her and they couldn’t, so eventually they hired a car, and brought the patient to the clinic. So, when they got to the clinic the nurses quickly ran, stabilised the patient, they were waiting, and they were waiting, put up magnesium sulphate, and the fits gone. And they came back and said, “what did you do, we spent the whole night trying to do that, what did you do?” and the people took the opportunity of then sitting down with them and then explaining to them that these are not demons you know and at that time we formed a group that we called For the Love of Women. If you love the women, you bring them you bring the women to clinic and we manage them. And they were very good, it worked so well they will bring pregnant women having their blood pressure checked and all making sure things were ok”.*

(Midwife, Zimbabwe, IDI18)

Healthcare professionals across the settings discussed the importance of avoiding judgment when alternative care was sought, informing women of potential harm caused and considering strategies to engage more meaningfully with alternative practitioners. Innovative ideas for collaboration and building trust were described.

*“I don’t have to show my feelings. The only thing I should do is to respect her beliefs and treat her with what do best. Because judging what they believe in is wrong. But I will also tell her it is harmful for her. I will advise her on the benefit and harm of what she has done without judging her.”*

(Midwife, Ethiopia, M33)

*“I’m always trying to see how I could make all of us united for the benefit of the population. Because I think if I have the possibility, I will have in every hospital a service of traditional medicine because it could have a psychological effect. We’ve lost a lot of sick people because they’ve left the hospital to go to a traditional healer but if they see it on site it might make it easier, they can still go and see them here at the hospital.”*

(Doctor, Haiti, IDI12)

Women, community members and health professionals suggested strategies to further improve access and quality of care. CHWs across the settings were often described as facilitators, educators and mobilisers helping to overcome barriers to facility-based care. Many CHWs reported their need for refresher training and further in-depth education to empower themselves with the tools and skills required to provide better services to pregnant women. In Ethiopia, there was a recommendation to extend the community health programme with more HEWs working in urban areas. In Haiti, health professionals felt education and incentivisation of TBAs were key to encouraging women to engage in facility-based services. Nurses requested further training on the management of hypertensive disorders in pregnancy. Community participants discussed low literacy as a barrier to health education, as well as lack of access to mass media and challenges with electricity supplies. Community participants discussed the power of using survivor stories for education as well as considering humour and humility to engage audiences. In Zimbabwe, HCPs felt a transparent complaints procedure at facilities, encouraging accountability and feedback were important. They emphasised the need for further training, including on the management of eclampsia and prescribing antihypertensive during pregnancy. Community meetings and engagement with churches were suggested as strategies to improve maternal health education. Discussion about the use of mass or social media included views that there was a potential lack of trust in these as methods of engagement. Other suggestions included engaging with alternative organisations such as a national association of traditional healers.

## Discussion

Despite the distinct sociocultural and health contexts of our study settings, the commonalities in conceptualisations of pre-eclampsia and care seeking, were striking. Women previously affected by pre-eclampsia described uncertainty and doubt related to their illness and for some, this was exacerbated by a lack of manifestations of ill-health. For others, care was not availed until serious complications had presented and the families of deceased women reported delays in reaching biomedical care. The variable and unpredictable nature of pre-eclampsia challenged collective understanding. The concept of an unseen or asymptomatic illness existing as part of the same entity as that which leads to convulsions, was not widely reported. Across all settings, there was the lack of a commonly used term equivalent to pre-eclampsia in local languages. Instead, various descriptive or interpretative terms were used to illustrate symptoms or meanings. We found across the three countries many women and wider community members attributed hypertension in pregnancy and its complications to diet, emotional or social distress and spiritual manifestations. Less commonly pre-eclampsia was understood to be an illness related directly to pregnancy. These findings were consistent with studies in other settings [15–18, 36]. Although regional nuances are seen with regards to perceptions of causes, similar themes are apparent across contexts. The common association of pre-eclampsia with psychosocial problems has the potential to be either protective or harmful to the pregnant woman. An attribution to stress or anxiety may lend itself to social protection and support of women, with the avoidance of strenuous physical labour and additional family care. However, where problems are related to perceived infidelity or marital conflict, the women or family may be stigmatised. The families of deceased women also described shame associated with the community’s perceptions of their circumstances. Future work could consider the use of participatory methods in destigmatising maternal and perinatal deaths through developing shared understanding of causes and community actions to reduce future risks.

Pluralistic care seeking was driven by different interpretations and attributions but also by social norms and expectations that changed over time. Divergence across the contexts centred around the nuances in religious or traditional practices relating to maternal health and pregnancy. Religion was an important influence shaping care seeking behaviours particularly in Zimbabwe. Women within some conservative Apostolic faith groups are subject to strict doctrines which may prevent them accessing facility-based care during pregnancy. A confession of sins during childbirth was described by some faith healers as a preventative or curative action. In communities where medicalisation of pregnancy and birth symbolises a lack of faith, engaging with religious leaders sensitively and building trusting relationships is crucial.

The autonomy of women was challenged not only in relation to particular religious practices but also with regards to financial control. Despite many women across the settings, describing agency to make decision regarding their care, they also reported requiring financial support from their husbands. Interventions focusing on involving male partners in maternity care have been shown to improve antenatal and postnatal care attendance, institutional delivery and complication readiness [37, 38]. Proposed mechanisms of action include increased support to overcome financial barriers, reduced fear of partner’s disapproval and men leveraging their own mothers to support women thereby avoiding potentially harmful traditional practices such as supplementary feeding of newborns with water or herbal infusions [38].

The hazards of using traditional treatments were reported by pregnant women and communities but there remained a social expectation to engage with them in certain circumstances, often driven by older women or mothers-in-law. Male involvement has also been associated with changes in couple’s communication and more equitable decision-making. However, some interventions may reinforce unequal gender relations, discriminate against single women or lead to delays where male involvement is interpretated as an obligation and attendance restricted by partner availability. In a study from Rwanda, women attending antenatal care alone were not consulted or were put last in line, and some single women hired or asked other men to pretend to be their partner [39]. Ensuring women are not pressurised to involve men if this is not their wish and avoiding performance-based incentives may help to safeguard against this [38]. The autonomy paradigm has been described as inadequate to explain gendered influences on maternal health, placing undue emphasis on individual action and failing to appreciate kinship structures and other influential actors such as older women [40]. Many participants in our study described shared decision-making with their partner or family and this should be considered in future maternal health education or behaviour change interventions. Valuing community systems and the nuances of social relations in different contexts must be considered to support pregnant women.

Quality of maternal care and equity are emerging as greater challenges to improving outcomes than insufficient access to services. Provision of quality care includes positive user experience which can improve retention in services, adherence to treatments and confidence in health systems [41]. Lack of respectful and compassionate maternal care has been widely reported [42, 43]. These barriers related to the third delay, or quality of care, are also likely to influence the first delay, or decision to seek care. Poor quality communication, health education and counselling disempowers women and impedes their ability to seek help appropriately [44]. Educational barriers around biomedical knowledge of pre-eclampsia were found in both HCPs and the wider community and were linked. We found health workers from facility to community level understood and taught that pre-eclampsia was caused by diet and stress. Whilst not backed up by current evidence, this may not be harmful and could potentially be beneficial in terms of reducing future risk of cardiovascular disease associated with pre-eclampsia.

Across our study settings, health systems relied on community level cadres to educate pregnant women and communities. This diverse group had varying levels of access to training, supervision and renumeration. Community health programs were developed to address gaps in equitable access to services for rural populations. CHW are familiar with the conflicts women face regarding competing priorities and agendas within families. However, their credibility and legitimacy rely on supportive systems within which they can function effectively [45]. In Ethiopia, WDG and HEW play important roles in educating and facilitating women to access biomedical care, but many have low levels of education and expressed a need for further support and training. Ethiopia has a clearly structured community health program; however, it relies heavily on volunteerism (WDG). The positions of CHW, who are often impoverished women themselves, should be considered in national and international policy, to ensure historical and current inequalities are not exacerbated [46, 47].

In Haiti, many women deliver without a skilled birth attendant. TBAs may be preferred by some women because they offer psychosocial support and attend to sociocultural needs. Payments may be in kind, negotiated or flexible. There is a lack of robust data on TBA practice and outcomes in Haiti. However, the health system is currently inadequate to support access to facility delivery for all women. TBAs’ practice is unregulated and training predominantly informal, with short-term donor-funded programs in some areas. Current global health policy is focused on institutional delivery and no longer supports TBAs. In some contexts, punitive measures are in place for those found to assist pregnant women at home. Previous studies have reported positive attitudes towards the role of TBAs during pregnancy [23]. Efforts to strengthen links between formal and informal health systems with the inclusion of trusted community level workers could reduce delays and negative impacts of pluralistic care seeking.

Our data showed that seeking alternative care was not necessarily in conflict or competition with biomedicine but could be complementary and used concurrently where systems allowed. Research examining collaborative models of care between traditional and faith healers, and primary care workers for psychosis has shown the approach was acceptable, effective and cost-effective [48, 49]. The model of partnership was thoughtfully designed, to respect traditional care whilst reducing harmful practices and supporting a rights-based approach. Guidelines for collaboration between alternative and biomedical practitioners exist for other illnesses and could be used to inform partnerships for maternal health [50]. Cultivating transparency and a bidirectional exchange of experiences can promote respect and foster trust. Acknowledging different philosophies and considering complementarity such a shared sense of responsibility for patient wellbeing, can also aid successful collaboration. In relation to pre-eclampsia, recognizing the disease-focused care offered by biomedicine and the holistic benefits of traditional or faith healing, could be considered.

### Strengths and limitations

This study focused on pre-eclampsia with the aim of understanding conceptual challenges in relation to help seeking. However, we recognise that many wider systemic and structural barriers influencing pre-eclampsia care exist and were not examined as part of this work. We included broad perspectives from multiple stakeholders across three distinct settings. The inclusion of facility-based health professionals alongside women and wider community members, contributed to a holistic understanding of community perspectives and exploration of factors relating to quality of care. Our research team was also diverse and interdisciplinary, offering a range of experience and positionality. Conversely, the breadth of participants included in our study may have limited our ability to engage deeply in understanding and representing each individual perspective. Data collection was conducted in local languages, however nuanced meaning may have been lost in translation into English. We recognise that within each country there are diverse sociocultural and economic factors at play and our study settings may not be representative of countries as a whole. However, our findings suggest many commonalities exist across distinct contexts, in relation to the barriers and facilitators relevant to pre-eclampsia care and help-seeking.

## Conclusions

The complexity that characterises pre-eclampsia is a challenge globally. Embracing the pluralistic nature of maternal care and pursuing a dynamic, culturally competent approach, grounded in idiomatic language and facilitating shared meaning, may help to address some contextual challenges relating to pre-eclampsia. The significance of faith-based and traditional healers to pregnant women and communities and their influence on care-seeking must be acknowledged and understood. Working collaboratively with different types of care providers could hasten improvements in maternal health and reduce preventable deaths due to pre-eclampsia. Further maternal health education efforts should seek to engage health professionals, partners, parents, religious and traditional healers, TBAs and the wider community, keeping the pregnant woman at the centre and aiming to empower her with knowledge and support.

## Supplementary Information

**Additional file 1.**

## Data Availability

The data generated and analysed during this study are not publicly available to protect individual anonymity but may be available from the corresponding author on reasonable request.

## Abbreviations

LMIC: Low- and middle-income countries
MMR: Maternal mortality ratio
TBA: Traditional birth attendant
HEW: Health extension worker
WDG: Women’s development group
IESO: Integrated emergency surgical officer
CHW: Community health worker
IDI: In-depth interview
FGD: Focus group discussion
SLT: Social learning theory
HBM: Health belief model
