# Supplementary material for: Pills and prayers: a comparative qualitative study of community conceptualisations of pre-eclampsia and pluralistic care in Ethiopia, Haiti and Zimbabwe
Source: BMC Pregnancy Childbirth. 2021 Oct 26;21:716. doi: 10.1186/s12884-021-04186-6 (PMC8547033; doi:10.1186/s12884-021-04186-6)
Supplement: Supplementary file 1 — Additional file 1. [file 12884_2021_4186_MOESM1_ESM.docx]

**Appendix**

1. Template Analysis: Health Belief Model & Social Learning Theory

| **CORE CONSTRUCTS** | | **HEALTH BELIEF MODEL** | | | | |
| --- | --- | --- | --- | --- | --- | --- |
|  |  | **Perceived Susceptibility** | **Perceived Severity** | **Perceived Benefits** | **Perceived Barriers** | **Cues to Action** |
| **SOCIAL LEARNING THEORY** | **Cognitive**  **(knowledge, expectations, attitudes)** | - Community awareness - Individual awareness - Danger signs - Risk perception | - Local knowledge and understanding of causes of complications - Understanding of risk of complications |  | - Quality of care |  |
|  | **Behavioural**  **(skills, practice, self-efficacy)** |  |  | - Biomedical help-seeking - Religious help-seeking - Traditional help-seeking | - Decision making - Experience - Skills and training - Self-efficacy |  |
|  | **Environmental**  **(social norms, access in the community, influence on others)** |  |  | - Medical pluralism | - Educational - Financial - Geographical - Material - Political - Sociocultural - Transport - Relationships with and between caregivers | - To improve access to information - To improve quality of care - To improve provider education and training - Preparations for birth |

1. Alternative treatments described

|  | **Treatments for hypertension in pregnancy** | **Treatments for seizures in pregnancy** | **Religious healing** |
| --- | --- | --- | --- |
| **Ethiopia** | Garlic, boiled cabbage leaf (*shiferaw*) | Lemons  Slaughtering of animals as a sacrificial offering | Holy water  Prayers (*Dua*) |
| **Haiti** | Almond leaf tea - drinking or bathing in  Sitting under an almond tea  Papaya leaf tea  Walking on hot soil to reduce oedema | Stripping clothes from pregnant woman, burning of clothes  Drinking water |  |
| **Zimbabwe** | A traditional non-alcoholic drink (*mahewu)* made of fermented maize meal and sorghum and taken with porridge  Avocado peel/stone boiled with water  Garlic  Snuff - smoking, bathing, drinking (taken by the healer before giving prayers)  Chewing raw groundnuts  Traditional beer or lots of water  Warm water and lemon - for “BP of the bones”  Eating caterpillars |  | Holy water and holy oil for protection or healing - may be drunk as an adjunct to prayer  Holy stones |
